# Delphi Prospection on Additive Manufacturing in 2030: Implications for Education and Employment in Spain

**DOI:** 10.3390/ma11091500

**Published:** 2018-08-22

**Authors:** M. Puerto Pérez-Pérez, Emilio Gómez, Miguel A. Sebastián

**Affiliations:** 1 Manufacturing and Construction Engineering Department, ETS de Ingenieros Industriales, Universidad Nacional de Educación a Distancia, Calle Juan del Rosal, 12, 28040 Madrid, Spain; msebastian@ind.uned.es; 2 Mechanical Engineering, Chemistry and Industrial Design Department, ETS de Ingeniería y Diseño Industrial, Universidad Politécnica de Madrid, Calle Ronda de Valencia, 3, 28012 Madrid, Spain; emilio.gomez@upm.es

**Keywords:** additive manufacturing, delphi prospection, 3D printing, education, employment

## Abstract

The term additive manufacturing (AM) groups together a set of technologies with similar characteristics forming part of the Fourth Industrial Revolution. AM is being developed globally, as evidenced by the standards published by and the agreements between the ISO and the ASTM in 2013. The purpose of this paper is to anticipate the main changes that will occur in AM by 2030 as forecast by more than 100 Spanish experts through Delphi prospection performed in 2018. In this way, the areas, aspects, and business models with the greatest probabilities of occurrence are obtained. The need for technical experts with specific knowledge and skills requires changes to current training syllabuses. Such changes will enable students to have the profiles foreseen in these job trends. The encouragement of STEAM (Science, Technology, Engineering, Arts, and Mathematics) training through the introduction of AM in study plans may be an appropriate alternative. Finally, the consequences of the Fourth Industrial Revolution for the employment market and on jobs, particularly in Spain, are set out and the latest Spanish Research, Development, and Innovation (R&D + I) plans are summarized as the framework for the possible implementation and development of AM.

## 1. Introduction

“Additive Manufacturing is a process for joining materials to manufacture objects starting from 3D model data, generally layer by layer, as opposed to manufacturing methods based on the elimination of material and shaping” according to the definition given in the ISO/ASTM 52900: 2015 standard [1]; therefore, the term “additive manufacturing” (AM) groups together a set of technologies with similar characteristics in terms of manufacturing forms with the ability to generate parts starting from a digital design and without using additional tools, generally by applying material one layer after another [2]. What is currently known as additive manufacturing has been referred to by different names since the 1980s, such as Rapid Prototyping (RP), Rapid Manufacturing (RM), 3D Printing, Rapid Tooling, Generative Manufacturing, eManufacturing, Constructive Manufacturing, Additive Layer Manufacturing (ALM), Direct Manufacturing, Direct Digital Manufacture (DDM), Freeform Fabrication (FFF), and Solid Freeform Fabrication (SFF), among others.

AM is not a single technology but a multitude of different technologies [3,4,5] forming part of what has been called the Fourth Industrial Revolution [6] and currently undergoing great development, some in the research phase and others sufficiently mature to be used in the manufacturing of finished products, i.e., products ready to be used [7,8]. AM is being developed globally as attested to by the standardization activities [9,10,11,12], the agreements between the ISO and the ASTM in 2013 [13], and the standards published by both the ISO [1,14,15,16,17,18] and by AENOR [19], indicating the importance of these technologies and the intention of standardization bodies to organize them in view of their tremendous potential. Current standards provide a classification of seven “process categories”. The ISO has still not published any specific standards on technologies and, although it is working on this, the difficulty is easy to understand, since some of the said technologies are still in the development phase and have not yet achieved maturity [20]. In different ways AM will transform manufacturing processes as we currently know them. Changes in supply chains and logistics [21], in business models [22], in the resulting products (embedded electronics, multiple materials simultaneously, one-off parts, short runs, previously impossible geometries). Also changes in the sustainability of manufacturing [21,22,23] and the behavior of the users (now converted into designers and producers or “prosumers”, i.e., a combination of both customer and supplier [5,24]), Will also change productwarranties [25]. Finally AM may imply major changes for the economy [4], society, and industry of the future [26].

The purpose of this paper is to anticipate the main changes that will arise in AM by 2030 as forecast by more than 100 Spanish experts through a Delphi prospection [27,28,29,30,31,32] conducted in 2018. The conclusions of the survey enable a likely development situation for these technologies to be sketched in for 2030, obtaining conclusions about the aspects or areas with the greatest likelihood of growth and development. The results confirm that Spanish experts believe AM will be a reality in manufacturing settings, optimizing processes and improving the sustainability of manufacturing, but that it will also take place in homes. The predominance of certain groups of technologies over others is posited. The strengths of AM and the challenges—not only technical—to be overcome are classified. The need for competent personnel with specific skills and know-how within this timeframe and the track record of experiments performed regarding teaching combined with AM [5,33,34,35,36,37,38,39] enable the advantages of including AM in syllabuses to be set out from primary education through to university-level courses.

## 2. Methodology of the Study

AM is a suite of disruptive technologies [40,41] that are transforming or complementing the world of manufacturing. The speed of their development and their latest applications in the most innovative sectors (vehicle manufacturer, aeronautics, and medicine) in opposition to the different levels of development between the various technologies and how this may affect different marketing areas raise the underlying question of this paper: How do Spanish experts believe that AM will develop over the decade ahead?

In consequence, the year 2030 was chosen as the time horizon as this was understood to be a sufficiently close period of time to enable the experts to make projections with solid criteria and also sufficiently far away for them to be able to suggest and implement conclusions that could be inferred from the results of the Delphi study (for example, suggestions regarding training).

In order to carry out a prospection on the future, it was decided to use the Delphi method, a procedure backed by its use in numerous scientific prospections and particularly useful when no objective information is available or in the face of situations of uncertainty [32,42,43,44,45]. According to Landeta [46], the main features of this method are anonymity, controlled interaction and feedback, group response, and the heterogeneity of participants.

This methodology consists of drawing up a survey around the problem formulated and sending it to a group of selected experts (round one). The results of the prospection are obtained using the set of responses as a whole. The same survey is then sent out again to experts who participated in the first round so that they can respond for a second time, but they are informed on this occasion of the overall results obtained in round one. Thus, participants can evaluate how close/far away each of their assessments are with respect to the average assessment of the population consulted and reconsider the response in round two, either by sending in the same option that they chose in round one (confirmation of the initial opinion) or else accepting the option of the average of the population as their own (changing their initial response to accept the group average) or bringing their initial response closer to the group response but without coinciding with it (influence of the group on individual response). The responses obtained generate a new set of consultation results with greater convergence in the responses. The Delphi methodology does not limit the number of iterations that could be applied but in the case we are concerned with, two rounds have been conducted, after which the results presented here were drawn up.

**Figure 1:**
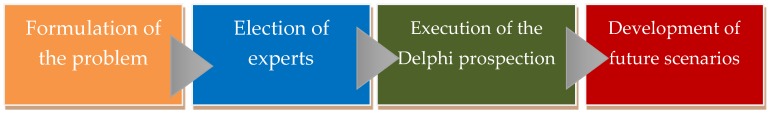
Main phases in the Delphi process.

Once the methodology to be used in the consultation had been pinned down, the work on drafting the questionnaire and the choice of experts began according to the four main phases in the Delphi process [47] as shown in Figure 1.

The target population for the survey is made up of researchers and lecturers in areas related to manufacturing engineering and materials engineering. The heterogeneity required is achieved by bringing in experts from all over Spain, with different responsibilities in a variety of geographical areas.

In order to facilitate contributions and to be able to achieve a relevant level of response, it was decided to limit the form, format, and extension of the consultation. The conditions agreed by the authors were as follows:

Consultations must be brief, concise, and set out without any additional explanation or clarification notes. The drafting of the survey must be straightforward. Concision in responding to the form was considered a key factor in order to be able to obtain a good level of response. The form was designed in such a way that the responses could be given with a single action by participants, without the experts keying in any data or values, for example, by clicking with a mouse. In order to unify the responses, the evaluation of each question had to be qualitative, rather than quantitative (the transformation of qualitative results to quantitative values was done by the research team after compiling all the individual questionnaires). The options available to participants were always an even number, in order to prevent the existence of a central option that might provide comfort for an uncommitted choice. It was agreed that the number of possible answers would be four, giving the experts the option to choose how much they were in favor of the future projection presented: not at all, partly, mostly, or fully. The consultation had to be kept short, enabling an expert to complete it in less than five minutes. For this reason, it was agreed that the survey would present between 15 and 20 scenarios. In the end, the survey was sent out with 21 questions.

**Figure 2:**
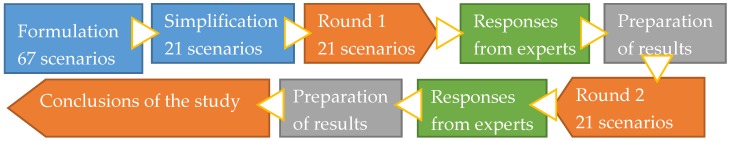
Main steps in the prospection process.

In the study, participants had to respond to the questionnaire individually, without knowing the answers given by the rest of the participants. Group data were shared with all experts only after round one was completed. The experts then decided whether or not the group response influenced them in their subsequent assessment. It was decided not to use the real-time application introduced by Gordon and Pease in 2006 [48] in which participants in a prospection could see the responses of the group of those who had responded previously, so that this information could influence their response (even in round one) [49]. Figure 2 provides a graphic outline of the steps in the process followed by the Delphi prospection.

The tool selected for presenting and sending out the consultation was Google Forms [50] because, in addition to being free of charge, it enabled the results to be sent to Excel immediately, thus facilitating the subsequent processing of the results. Email was used for communicating information [51].

### 2.1. Progress of the Main Stages in the Study

#### 2.1.1. Formulation of the Problem

As indicated above, the survey comprises 21 scenarios, the result of an effort in summarizing by the research team which initially started with 67 questions including various aspects and perspectives at first considered relevant [52]. The formulation of the survey covers the most significant aspects of the initial compilation and is divided into three distinct blocks: The first contains questions about the market, business models, supply chain, new products, services and applications, specific training, certification, intellectual property, and warranties. The second block attempts to view the process categories that will be used in 2030 depending on the manufacturing models. The third block focuses on the possible manufacturing technologies that will prevail, and on the strengths and weaknesses of AM.

These 21 scenarios were tested and adjusted by the research team as specified in detail in Table 1, Table 2 and Table 3.

#### 2.1.2. Selection of Experts

**Figure 3:**
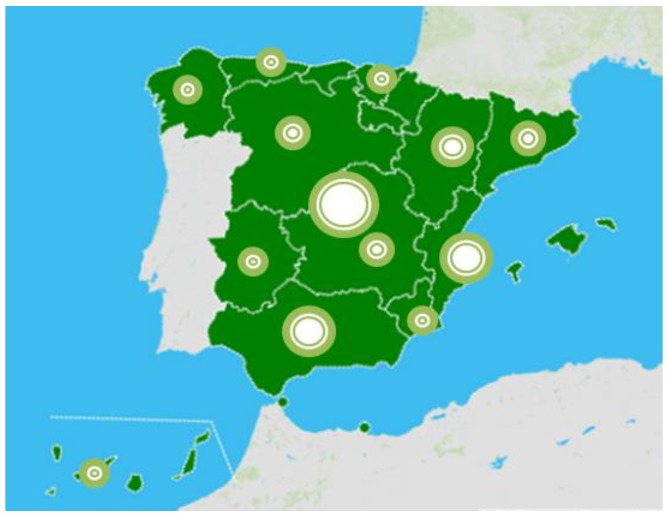
Participation of experts, graphic view based on the location of their centers or universities.

The experts invited to take part in the study are mostly Spaniards and belong to universities, technological centers, or institutions related to manufacturing engineering and/or materials engineering, basically lecturers and researchers. The participation of the experts, classified according to their geographical location, is shown in Figure 3. A high concentration in Madrid is appreciated due to the large number of experts consulted in the universities of its geographical area.

Of those who finally decided to complete both rounds of the survey, 21% are women. In total, they represent 31 centers and universities from 15 different regions of Spain.

#### 2.1.3. Execution of the Delphi Prospection

The questions were posed following a different structure depending on the block that they belong to. In the first block it was possible to assess the position of the experts’ proximity to the scenario proposed on a scale of 1 to 4 from “not at all” (total disagreement) to “fully” (total agreement). Only one of the four options could be chosen. In the second block, the questions were posed using a matrix, as it was intended to assess which AM process category (as defined in ISO standard 017296-2: 2015 [16]) will adapt more to the two different extreme types of manufacturing: home-based manufacturing and hybrid manufacturing. In the third block, it was necessary to choose three options from those proposed, with the expert being allowed to add another one if considered appropriate.

## 3. Results and Discussion

The results obtained are set out in the following three subsections. Comments and questions raised by the said results are also included.

### 3.1. Results of Block 1

In the first block of questions, a consensus of 50% or more was reached in 15 of the 16 scenarios proposed.

It should be highlighted that there was more than 70% of coincidence in the responses received, indicating clear agreement among participants. Of these, 77% felt that conventional lathe manufacturing will not cease to be used in the timeframe considered (2030). This question was intended to find out whether, in view of the developments and trends in AM, the experts felt that by this time a radical change would have taken place in manufacturing methods with respect to how items are manufactured at the moment, using the lathe-turning process as the most representative. A majority response in the affirmative would indicate the need for drastic changes in the training of students, so that those completing their studies in 2030 would be prepared for the radical transformation in manufacturing techniques suggested by the hypothetical response. Since the response has mostly been one of disagreement, it is inferred that current syllabuses must continue to include traditional manufacturing methods and that the changes necessary in view of the progress in manufacturing methods can be sequential.

It is also interesting that only 7% of survey interviewees felt that more than 50% of production would take place using AM technologies in 2030. In total, 94% believe that this situation will not arise or will occur only on an occasional basis, strengthening the idea that AM will end up being integrated into the current manufacturing systems without disruption (except perhaps in highly localized niches such as medicine where new and very advantageous opportunities arise with the personalization of prostheses and organs, for example [53,54]). The panorama set out is perfectly compatible with the manufacture of very short runs or single items, perhaps customized for the user. In fact, 63% of the experts felt that this niche of personalized items will be an inherent characteristic of AM technologies and that AM will be the exclusive means for their manufacture. AM techniques can, of course, be applied to personalized manufacture without any increases in cost (except in the design part), time, or effort, implying an immense differentiating advantage.

Of the participants, 54% fully agree with the statement that “In 2030, more than 50% of products will be manufactured at factories where AM is included among the manufacturing processes as just another group of technologies”.

The responses mentioned above indicate that the future manufacturing model will include AM as another group of available technologies, for use whenever convenient or efficient for the processes, which will be combined and used by those responsible for manufacturing to produce as efficiently as possible. In this model, AM technologies will be integrated and used in factories just like any other current technology.

It is also indicative that 89% of experts believe that domestic manufacture of parts will be an occasional or majority reality by 2030, but there is dispersion regarding the opinion that more than 50% of households in the industrialized countries will own this technology (perhaps because of the technical requirements needed? Because of the lack of guarantee for the items manufactured? Due to the scant application they foresee? Because their use in households at the moment is mostly among amateurs?). According to the results obtained from the survey carried out to the experts, the least likely marketing model is that of a specialist store close to consumers, which may recall the model of high-street photocopying and printing centers that were so successful in the 1990s and where the work would be carried out, or at least advice provided, by a specialist technician.

One of the main challenges for the future development of a market for one-off or customized parts manufacturing is being able to give a warranty for their manufacture. How can a warranty be issued regarding a part with a unique design? Any design change with respect to a standard part may affect its aesthetics, functionality, features (its resistance or conductivity, for example), or any other aspect of the product. How can this problem be dealt with in a generalized manner? Responding to this question is key for the customized manufacturing niche to be able to grow. A 76% majority of participants believe that these issues will be resolved by 2030.

In addition, 71% of those surveyed state that AM technologies will for the most part be classified and standardized by 2030. For this to happen, the current panorama needs to be clarified: technologies need to be developed and documented prior to their subsequent standardization. The standardization of AM is currently underway, partly due to the dizzying advances in techniques, materials, uses, and other characteristics of these technologies. The current standardization status is advancing according to the work carried out by the ISO TC 261 Technical Committee [55].

How will the application of AM technologies affect the sustainability of manufacturing in 2030? Will it help to increase sustainability of made-to-order manufacturing by lowering levels of raw material and stocks in storage, or reducing the consumption of fuel for transportation because items are manufactured closer to their end users? On the other hand, will the manufacture of single parts or small runs mean that the energy synergies of serial manufacturing will be lost and more energy will be consumed? Of the responses, 78% indicate that AM will contribute mostly or fully to the sustainability of manufacturing. Obviously, this paper is not sufficiently extensive to be able to delve deeper into this issue, but this is a very interesting aspect for possible future research [56,57].

Each AM technology requires specific qualifications although, taken together, they all share a preparation in digital design techniques. If specific qualifications are going to be required in 2030 as almost 90% of participants believe either mostly (62%) or fully (27%), it is necessary for universities and technical training centers to apply changes to their syllabuses. These changes must begin as soon as possible so that the technical experts are ready by 2030. The definition of the changes required to current syllabuses is, of course, outside the scope of this study, although the skills that will be necessary in the new digital period are suggested in the last part of the document.

Of the experts, 69% believe that AM will be used occasionally to manufacture tools in contrast with 58% of them who believe that more than 70% of prototypes will be manufactured using AM technology 2030. This is an interesting differentiation from which it may be inferred that the immediacy of AM is seen as a clear advantage for prototyping, preliminary models, use in marketing, etc.

By 2030, the tools and control devices capable of monitoring and overseeing real-time production using AM techniques will have been developed. In total, 90% (61% + 29%) of interviewees believe mostly or fully that this will be a reality. Monitoring of processes simultaneously with manufacture rather than after manufacture is complete is one of the challenges currently facing AM for use as a standard manufacturing technique. Control is currently possible but it takes place after the part has been finished, requiring the post hoc rejection of any not meeting specifications (with the evident loss of resources). Real-time monitoring has the advantage of increasing the degree of specification-compliant manufacturing. The experts consulted mostly believe that this integration will arise in 2030, indicating that the development of control techniques and sensors are fields with extensive future scope for development.

With respect to the supply chain, 60% of interviewees feel that the distribution of designs for AM will in general be done digitally, using one or more databases. Of the experts, 23% believe that this will be a reality. In other words, there is a consensus among the experts that designs will mostly be acquired from a digital market accessed by users. This confirms the theory in some publications stating that there will be two markets: one for the marketing of digital designs and another for the marketing of manufactured items. Some of these theories maintain that production values will become digital, as has already happened in other sectors (telephony and Internet). That is to say, the value of the product will lie more in its digital part (design, in this case) than in the physical manufacture of the product itself; therefore, the “value” of the design (the digital purchase) will be far higher than that of physical manufacture, which will end up being a “utility”. Another question arises in connection with this one: we wanted to know whether the experts felt that that digital market would be freely accessible (following the current trend for collaborative models) or whether payment would be required (thus monetising part of the supply chain). Most of the interviewees feel that the design market will be a pay-to-use market and will only occasionally be free of charge (or collaborative). The following questions arise immediately: Who will control this market? Large manufacturing corporations, the manufacturers of AM technology, or the users designing the models? Or perhaps it will be none of these and only large digital platforms such as Google or Amazon will be capable of reaching users?

Regarding the supply chain last questions, 61% responded that the distribution sector is going to undergo a transformation towards delocalization as confirmed in the work by Sray et al. [58]. Of the experts, 55% mostly feel that “more than 50% of manufacturing will be delocalized or distributed” and 6% believe that that scenario will be a reality. This question explores the possible changes that may arise in the distribution sector. A large part of centralized mass manufacturing models corresponds to product logistics and distribution. Manufacturing close to where assumption takes place would alter the weighting of the different segments in the production chain. Distribution, currently the key to mass production systems, may lose its relative importance in future, probably giving rise to a transformation in order to serve users differently than at present.

**Figure 4:**
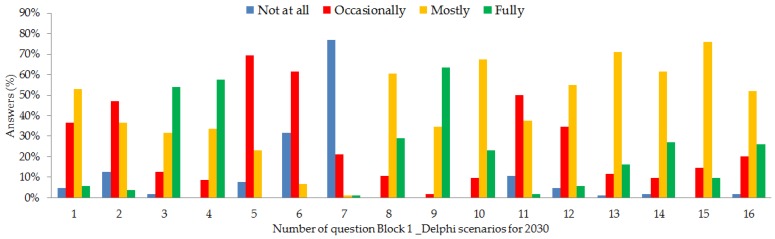
Results of Block 1 questions (%).

The numerical values for the results obtained are shown in Figure 4.

### 3.2. Results of Block 2

As mentioned above, the two questions included in this block tried to glimpse the process categories (according to the classification in the ISO 17296-2 standard [16]) that will apply in each of the two extreme manufacturing models: domestic manufacture and hybrid manufacture (in factories).

According to the participants consulted, domestic “printers” based on “material extrusion” AM technologies are most likely to be implemented in homes.

However, “material projection” and “localized energy deposition“ technologies (76% including both “mostly” and “fully”) followed by extrusion technologies are those believed by experts to be in most widespread use in hybrid manufacture by 2030 (in use just like any other technology in factories). Sheet lamination and photopolymerization technologies will be used less in manufacturing centers, according to these results.

**Figure 5:**
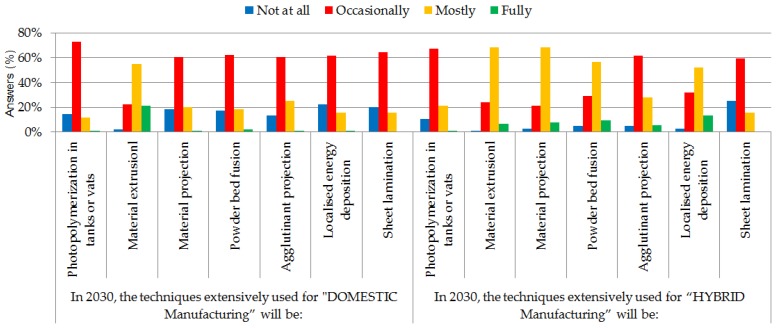
Results of Block 2 questions (%).

The numerical values for the results obtained are shown in Figure 5.

### 3.3. Results of Block 3

In this set of questions, we were trying to consult opinions about which technologies will lead the field in 2030, and also discover the three strengths of AM and the three most important challenges to be resolved.

According to the specialists, the technologies that will prevail over others in the market in 2030 are fused deposition modelling (FDM), selective laser sintering (SLS), and selective laser modelling (SLM), as shown in Table 4.

The results indicate that the most relevant factors for the development of AM are related to the product itself and to production and market factors, as shown in Table 5. Design freedom gives the possibility to manufacture products with as-yet-impossible geometries. In addition, flexibility in design changes gives the ability to create different products with the same base, thus generating infinite possibilities for personalization, adaptation, etc. Secondly, the experts are of the opinion that being able to produce short manufacturing runs or one-off items (personalization) is a determining factor for AM to take off. Personalized manufacturing is a unique feature of AM technologies that does not make the process more expensive, except in the digital part, since each item requires its own design which will not be reused. Lastly, the reduction in development time and time to market is the third most voted characteristic. This quality enables items produced with AM technologies to be used for the verification of a product’s operation on the market with possible savings in terms of time and cost with respect to current procedures, a matter of great value for developers and promoters.

The technical limitation for achieving the properties required in the end product stands out among the other challenges to be overcome. Anisotropy [59] and the control of physical properties (such as resistance to traction, size verification, or the surface finish), of chemical properties, or of any other kind of property required in the product are clearly an essential current limitation for the use of AM technologies beyond one-off developments or highly specific market niches. In addition to the improvement in the control of products’ properties, the experts believe that there is currently a limitation on the manufacturing process (a limitation on manufacturing volume, mainly due to the manufacturing equipment, production speed, or a combination of both factors). The latest developments from equipment manufacturers try to resolve the speed limitation by placing multiple heads that work simultaneously. Lastly, the certification and warranty of parts and finished products is the third important challenge receiving the most votes from the participants interviewed, as shown in Table 6. It is useless to manufacture the desired part to the required specifications and at a suitable speed if, at the end of the day, it is impossible to certify what has been produced. Certification is one of the constraints currently found in sectors like aeronautics [60].

## 4. Implications for Education and Design of Training Programs

Several studies have been published on the development and/or implementation status of AM in different countries—USA (Minnesota) [61], Finland [62,63], South Africa [64,65], Germany [66], Mexico [67], India [68], UK [69], China and USA [70]—analyzing a number of aspects, an indication of the importance of these technologies.

The prospect of using information and communication technologies (ICTs) and massive automation (which might include, among others, the use of AM, smart software, robotics, drones, Artificial Intelligence (AI), or Big Data) ushers in the need for specialists to carry out the necessary research in order to overcome current challenges as well as technicians able to lead the technologies to a suitable level of maturity for them to be used on the market and, in particular, in the manufacturing industry.

In order to have human capital available with skills in these fields of optimization and ICTs, it is necessary to design a national-level training plan starting from basic education (primary schools) all the way up to university degrees, particularly in engineering [71]. The training needed is directly related to STEAM (Science, Technology, Engineering, Arts, and Mathematics) and ICTs.

The inclusion of the use of AM in current syllabuses is necessary if the opinions of Spanish experts turn out to be true and, in 2030, technicians with specific qualifications for AM manufacturing are going to be needed.

Education must provide the knowledge and skills for future professionals to be able to work in the future market, within the framework of the Fourth Industrial Revolution. Future technicians will be required to have cognitive competence in order to deal with a changing technological environment (mathematics, logic, data processing, project management). In addition, they will need to have noncognitive skills [72] (critical thinking, teamwork, achievement of goals, interpersonal relationship skills, or troubleshooting abilities [73]), bearing in mind that jobs requiring noncognitive skills will be the last to be replaced by automated processes and therefore will be among the most valued. The skills mentioned above should be transmitted through education, although some of these are difficult to measure and evaluate using conventional testing [74].

AM technologies require the use of different academic disciplines such as material sciences, machinery design, fluid mechanics, heat transfer, computing, statistics, graphic design, etc. These disciplines can be treated as and when practical manufacturing is developed, in order to help with their acquisition or a deeper understanding depending on the prior training of the students in question [34].

A number of different experiments have shown that the use of AM in educational processes speeds up the training and makes it more interesting, bringing education closer to the real world. Some experiments in the use of AM technologies in training at different levels of education and in multiple countries have provided an overview of their usefulness and the knowledge and skills developed through these practices.

There are several actions and experiments reported in the literature documenting the advantages of introducing AM into training, both for schools [33,75,76,77,78] and for universities [36,37,39,79], for teachers [38], national plans [69,80], and even in libraries [35]. These experiments have shown that, in addition to the disciplines mentioned inherent to manufacturing, participants also develop other nontechnical skills such as teamwork and collaboration, creativity, flexibility vis-à-vis changes, communication skills, handling of changing information, concentration, planning, perseverance, and self-control.

Although there is evidence that training through AM is very useful, the experiences documented to date have been carried out by professors or researchers using their own methodology. There is as yet no specific textbook or methodology for implementing this kind of training [5], yet, nonetheless, the conclusions of the practical activities documented show a positive result.

## 5. Implications and Consequences for Employment

The emergence of technologies in society, new consumer behavior, and the birth of new business models form the pillars of what is known as the Fourth Industrial Revolution [6,81,82].

AM is a group of technologies of great importance within the generic framework of the Fourth Industrial Revolution, since on the one hand it moves within the ICTs, but on the other it is clearly a productive resource that is largely alternative to the more traditional manufacturing processes (such as it emerges from the prospective study of the previous section), even to those processes with higher levels of automation. Therefore, in the field of future employment and its expectations, the increasing use of AM technologies will contribute to enhancing the strategic nature of ICTs and highly automated manufacturing, representing a niche for employment framed in both ICTs and in highly automated advanced manufacturing processes.

Technological transformation will affect employment and the welfare state in a manner that will not be homogeneous across industries, occupations, and countries [83,84,85,86,87,88,89]. Numerous authors defend the direct correlation between an increase in automation and the level of unemployment based on the idea that robots will increasingly be able to carry out tasks currently allocated to humans [88] as their dexterity increases, becoming more efficient and requiring no rest. The truth is that the Fourth Industrial Revolution is full of uncertainties; not only with regard to employment levels but also the quality and variety of the same, and also with respect to a variety of aspects such as business models, in-demand professions, displaced occupations, and those others that will emerge to offer new services, earned income, and legislation, to mention a few.

### 5.1. New Professions and the Disappearance of Some Current Occupations

Smart software, robotics, Big Data, Artificial Intelligence (AI), drones, and Additive Manufacturing (AM) will displace certain activities and professions and will lead to the development of other, new professions that do not currently exist [90,91]. The risk of job losses in Spain over the next 10–12 years as a result of automation (jobs with at least a 70% probability of being replaced by machines) is estimated at 12% according to the study The Risk of Automation for Jobs in OECD Countries [92], published in May 2018. According to BBVA Research published in March, 2018 [86], this figure is 36%. This result is based on a 2016 study by Frey and Osborne [88] which classified 702 different professions in the United States according to their chances of being substituted as a result of automation.

Several authors [92] put the results of the study by Frey and Osborne into context [88], arguing that although the rate of technological development is exponential (one of the characteristics of the Fourth Industrial Revolution), integration of such technology in society and industry does not depend exclusively on technological development factors but must be considered more holistically to take into account the political and social context of each country and must also consider the human factor; that is, new technologies that are developed will be taken on by society not upon their development but once society is ready for them.

**Figure 6:**
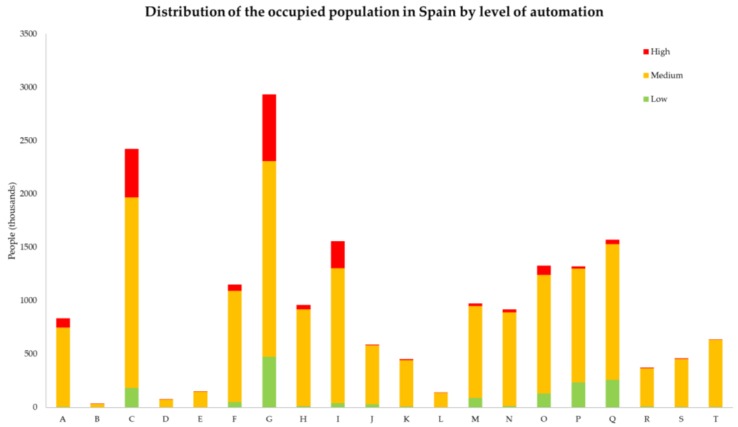
Probability of the distribution of the occupied population in Spain according to the level of automation. A Agriculture, farming, forestry, and fisheries; B Extractive industries; C Manufacturing industry; D Energy supply (electricity, gas, steam, and air conditioning); E Water supply and treatment, waste management; F Construction; G Wholesale and retail trade, motor industry; H Transport and storage; I Hotel and catering trade; J Information and communications; K Financial and insurance activities; L Real estate activities; M Professional, scientific, and technical activities; N Clerical activities and ancillary services; O Public Administration and defence, Social Security; P Education; Q Healthcare activities and social services; R Artistic, leisure, and entertainment activities; S Other services; T Domestic service activities in households.

Quantitative values help us understand the significance of this issue and which prevention measures are essential. By considering the automation probabilities obtained by Frey and Osborne [88] and applying them to the active population classified by economic activity in the first quarter of 2018 in Spain published by the National Statistics Institute [93], we find that 1,750,000 persons are employed in jobs with a high probability of automation (those with a probability above 70% of being replaced by machines), and only 1,550,000 are at a low risk of automation (with a lower than 30% probability of being ousted by machines). This information can be seen in Table 7 and is displayed graphically in Figure 6.

### 5.2. Digital Divide

One of the consequences of automation will be that new, as-yet-undefined jobs related to technology will emerge. These occupations will require specific training and skills that not all the population will have access to. Jobs with higher qualification requirements will be increasingly better paid and jobs with low qualification requirements will be increasingly worse paid. In all of these, routine tasks may be automated. This may give rise to the displacement in the outsourcing of routine tasks, which are currently outsourced to countries with lower wages and which, in the future, may be outsourced to machines and robots [6]. This situation gives rise to what is known as the “digital divide” between workers in the digital market and those outside it. We can think of it as a society with separate “castes” as a result of a highly polarized labor market (which is very dangerous for social stability).

As Frey and Osborne established [88], if we separate the labor market into three segments—highly paid, highly technological jobs (or with “human” requirements); average jobs (not very technological and not very routine work) with average pay; and highly routine work with very low wages—then those in the middle tier are the most prone to being replaced by automation as the cost/benefit ratio will be more profitable (sales, administration, transport, service, productive or manufacturing tasks). The first group requires high qualifications or characteristics that are difficult to automate and are related to human behavior (programmers, specialists in digital security, designers, psychologists, doctors, artists, teachers, judges, etc.); wages for the last group will be so low that the investment required in automation will not be worthwhile (maid services, delivery persons, farming harvesters).

Unlike previous industrial revolutions, even nonroutine jobs which are not currently considered as being open to being robotized will be open to being robotized in this so-called Fourth Industrial Revolution. This change will take place as the skills required are developed, such as sensorization, artificial intelligence, and massive storage in a super-reduced space (for instance, self-driving vehicles).

In order to carry out this training at all levels, including at business level, policies must be put in place to foster research development in all areas (university, business, individual, institutes of technology) and to promote reindustrialization to compensate for the loss in relevance of industry in favor of services in developed economies [94,95,96]. In short, implementing an effective employment policy [97]. It seems necessary to generate a structure that suitably fosters labor reintegration and lifelong training and guarantees equal opportunities [98,99,100,101]. In particular, the reintegration into the labor market of workers who missed the technological train and whose “digital divide” widens every day will require specific support and training to return to paid employment.

### 5.3. Labor Market

The labor market will change (and is changing) both in geolocation and in regulation. Globalization allows companies not to have boundaries. This has an effect not only on multinationals with headquarters in different countries [102]. Small and medium enterprises will also be global—many already are—since their services may be hired over the Internet.

Employees will work in a global market, leading to labor and trade union regulations that are no longer national in scope. There will be no territorial limits. Industrial relations will be ultraflexible in regard to both jobs and competences, and highly diversified: new regulations will be required to regulate this added staff flexibility (workforce on demand). Our grandparents worked for one company all of their lives; it is now normal to work for 3 to 7 companies in the course of a career; our children will simultaneously work for 7 different companies, probably carrying out different tasks. Companies will try to hire talented staff (Digital Talent) regardless of their place of birth.

The ties to a job or to a single company will weaken until they disappear, there will be constant changes in activity and/or employment, and many employees will not be required to go to work in an office. Employment will be a mixture between a paid job and self-employment [103]. This model is called the gig economy, collaborative economy [104], or freelance economy, using the Internet as a platform [105]. By way of example, we may consider the Uber model; namely, short-lasting jobs for specific tasks. Highly qualified professionals will work independently for several companies, organize their own time, and develop the skills needed to participate in multiple work teams [106]. In the gig economy, everyone is their own boss, setting their own working times (earnings permitting); however, workers are neither paid employees nor self-employed workers. Social protection for these workers must be developed accordingly. Workers will adjust to market requirements and, similarly, legislation must change to follow these labor models [96]. One of the possibilities is evolving towards “flexicurity”, which considers “the protection of flexible employment contracts and training and reintegration policies” [6], a model that Scandinavian countries have already developed.

Flexibility to carry out different jobs will be a valued skill, which leads to the need for continuous and permanent training throughout a worker’s professional life, affording them the quality of adaptation in such a way that education in the 21st century should be “teaching to learn”. “Education is closely linked to technological change: it isn’t only shaping the inventors of the present and future but is providing the entire population with the tools required to adapt to and make the most of new technologies” [74,107].

In order to mitigate the effects that the Fourth Industrial Revolution may have, public administrations should draw up and publish in advance a plan to eliminate institutional barriers and foster infrastructures minimizing any negative impacts on the labor market.

Investment in human capital (a production factor that depends not only on quantity but also on quality, the level of training, and productivity of persons involved in a productive process) will be an increasingly determining factor for economic growth [108].

To this end, teaching centers and universities must foster a more extensive and tighter collaboration between University and Business, especially in Spain where university research is not easily transferred to companies, which in turn leads to a low impact of financing in private research.

### 5.4. Investment in Technological R&D + I (Research, Development, and Innovation) in Spain

The development of AM and its use in manufacturing depend on culminating several research, development, and innovation processes. R&D + I investment in each country is a benchmark for the support given by government policy to fostering economic growth and productivity, and is understood as the basis for progress and social wellbeing in that country.

Below is a summary of the main plans and policies related to R&D + I in Spain.

In response to the Europe 2020 Strategy [109], Spain amended its “National Science and Technology Strategy” (2007–2015) [110] by passing the “National Innovation Strategy” (E2i) in 2010, with five priority axes [111].

In 2012, the “National Strategy for Science and Technology and Innovation 2013–2020” [112] replaced the earlier plans in reaction to the fall in R&D financing in Spain (changing the upwards trend of previous decades), partly as a result of the economic crisis, and after verifying that the results obtained by the two former plans had not reached the objectives set out.

The “National Plan for Scientific and Technical Research and Innovation 2017–2020” [113] is the instrument used by the General State Administration to implement the scope of objectives set out by the Europe 2020 strategy and the Spanish Strategy. This Plan only includes financial aid allocated to R&D and is made up of four State Programs (similar to the “National Plan for Scientific and Technical Research and Innovation 2013–2016” [114].)

The trend seen in the main indicators reflects the state of R&D in Spain. These indicators can be consulted on the FECYT website (Indicators of the Spanish System for Science, Technology and Innovation. 2017 Edition) [115].

## 6. Conclusions

The term AM groups together a suite of technologies undergoing development and framed within the so-called Fourth Industrial Revolution. The results of a Delphi prospection carried out with over 100 Spanish experts have highlighted that AM will be included within existing manufacturing processes and will change various aspects of manufacturing: From the business models and the distribution chain to the concept of client and supplier. The likeliest panorama in 2030, according to the responses from experts, includes the integration of AM as more efficient and more sustainable processes in factories and the domestic use of these technologies. The predictions indicate that many processes will be developed and classified in AM, and that certification and standardization will be a reality. AM will grow most in the prototype market, occasionally in the tools market, and will be of practically exclusive use in customized manufacture. AM has certain features that will boost its take-off, such as the possibility of shorter production runs, the capacity to manufacture parts with geometries that are impossible using current methods, flexibility with respect to design changes, or the prospect of generating unique items. For this to happen, AM has to overcome certain challenges, such as ensuring the quality of each phase of the production process (so that the properties of the products obtained in this way can be certified), the lack of homogeneity in product properties, or the limitation of current AM machines with regard to manufacturing volume or production speed. The groups of AM technologies that will be in greatest use in domestic manufacturing models and in factory manufacturing are specified. The manufacturing business model may change. The designs for parts will be housed in digital databases that will mostly be available on a pay-for-use basis, according to our interviewees. The distribution chain will be changed as and when changes arise in the size and location of production centers.

The result of the study considers a future in which AM is developed and integrated into manufacturing processes. For this transformation to be successful, it will be necessary to have competent technicians available and adequately trained to cope with the changes that will arise. The skills and know-how needed for this are still pending definition, development, and offering at universities and training centers. One of the possibilities is the use of AM itself as a training tool, through which not only is a knowledge of the many areas needed for the achievement of manufacturing communicated, but the students also develop noncognitive skills such as teamwork, change management, or the acceptance of errors, which will be highly appreciated in that new context. The use of AM also provides practical training that motivates and challenges students.

The use of AM, alongside other digital techniques such as smart software, robotics, Big Data, and Artificial Intelligence (AI), is part of the Fourth Industrial Revolution. This Revolution will entail a change in the job market, polarizing professions and occupations depending on whether or not they use these technologies. Some professions will disappear and others, as yet unknown, will become essential. Permanent training and the inclusion of STEAM as part of educational plans are presented as options to minimize these effects.

## Figures and Tables

**Table 1:** Delphi scenarios for 2030. Block 1 of questions.

No.	Block 1	Scope
1	In 2030, it will be possible to manufacture parts in more than 50% of homes in industrialized countries	Business model: Domestic/specialist store/large factory
2	In 2030, more than 50% of products will be manufactured in specialist stores with specialized personnel close to consumers (much like photocopiers in their day)	
3	In 2030, more than 50% of products will be manufactured in factories where AM is included among their manufacturing processes as just another group of technologies	
4	In 2030, more than 70% of prototypes will be manufactured using AM technologies	Development of real sectors in the market/changes in manufacturing
5	In 2030, more than 50% of tools will be manufactured using AM technologies	
6	In 2030, more than 50% of global production will be done using AM technologies	
7	In 2030, lathes will not be used for manufacturing	
8	In 2030, AM processes will be monitored in real time Sensors and production control devices will be integrated and widespread in AM	Quality assurance and part inspection
9	In 2030, there will be a new market niche for customized production runs that can only be manufactured using AM	
10	In 2030, users will obtain the digital formats of the parts to be manufactured from one (or more) databases	Supply chain and distribution
11	In 2030, digital formats will be free of charge and freely available	
12	In 2030, more than 50% of manufacturing will be delocalized or distributed. Production will take place at points close to the consumer and the distribution sector (supply chain) will have changed to service this new kind of manufacturing	
13	In 2030, more than 75% of AM processes and technologies will be classified, its production characteristics documented and standardized	Degree of AM maturity/qualification of personnel/legal
14	In 2030, specific training and qualification will be needed to produce using AM	
15	In 2030, there will be a procedure for issuing a warranty for unique parts (personalized) manufactured using AM technologies	
16	In 2030, AM will have contributed to the sustainability of manufacturing (manufacturing will be less polluting than at present)	Sustainability

**Table 2:** Delphi scenarios for 2030. Block 2 of questions.

No.	Block 2	Scope
17	In 2030, the techniques extensively used for “DOMESTIC Manufacturing” will be	Process categories
	Photopolymerization in tanks or vats	
	Material extrusion	
	Material projection	
	Powder bed fusion	
	Agglutinant projection	
	Localized energy deposition	
	Sheet lamination	
	Other	
18	In 2030, the techniques extensively used for “HYBRID Manufacturing” will be	Process categories
	Photopolymerization in tanks or vats	
	Material extrusion	
	Material projection	
	Powder bed fusion	
	Agglutinant projection	
	Localized energy deposition	
	Sheet lamination	
	Other	

**Table 3:** Delphi scenarios for 2030. Block 3 of questions.

No.	Block 3	Scope
19	Choose the 3 technologies that, in your opinion, will prevail over others on the market in 2030 (i.e., will be most used). Which 3 technologies will outlive the others?	Technologies
	Stereolithography (SLA)	
	Fused deposition modelling (FDM)	
	Selective Laser Sintering (SLS)	
	Selective Laser Modelling (SLM)	
	Direct Metal Deposition (DMD)	
	Laminated Object Manufacturing (LOM)	
	Other	
20	Indicate the 3 factors you consider most relevant for AM to “impose itself” on other manufacturing methods in 2030	Strengths
	Democratization of manufacturing	
	Freedom of designers. Flexibility for design changes	
	Reduction in product development cycles and time to market	
	Lower tooling costs	
	Shorter production runs. Customized or one-off production	
	Lower raw material costs (less waste)	
	Reduction in transportation and distribution costs and times	
	Reductions in storage: Of raw materials and finished products	
	Contribution to environmental sustainability	
	Other	
21	Indicate the 3 key factors you think must be resolved for AM to “take off” by 2030	Weaknesses
	The technical limitation for achieving the properties required in the end product	
	The certification of parts and finished products	
	Changes in the way of thinking when designing parts	
	Industrial property rights, taxation, and the safety of the products manufactured	
	The needs for training of AM equipment operators	
	The cost of raw materials, machinery, and/or transportation	
	Limitations on the volume and/or speed of manufacture	
	The need for post-processing	
	The integration of AM into current manufacturing methods	
	Other	

**Table 4:** Results on technologies. Block 3 (%).

AM Technologies	Prevalence
Fused Deposition Modelling (FDM)	29%
Selective Laser Sintering (SLS)	25%
Selective Laser Modelling (SLM)	25%
Direct Metal Deposition (DMD)	12%
Laminated Object Modelling (LOM)	2%
Stereolithography (SLA)	6%
Others	1%

**Table 5:** Results on the strengths of AM. Block 3 (%).

Factors for the Development of AM	Prevalence
Freedom of designers. Flexibility for design changes	28%
Shorter production runs. Customized or one-off production	25%
Reduction in product development cycles and time to market	21%
Reduction in transportation and distribution costs and times	6%
Democratization of manufacturing	5%
Lower tooling costs	5%
Reductions in storage: of raw materials and finished products	5%
Lower raw material costs (less waste)	3%
Contribution to environmental sustainability	2%
Others	0%

**Table 6:** Results on AM weaknesses. Block 3 (%).

AM Weaknesses	Prevalence
The technical limitation for achieving the properties required in the end product	28%
Limitations on the volume and/or speed of manufacture	21%
The certification of parts and finished products	19%
The integration of AM into current manufacturing methods	10%
The cost of raw materials, machinery, and/or transportation	7%
Changes in the way of thinking when designing parts	6%
The need for post-processing	4%
Industrial property rights, taxation, and the safety of the products manufactured	3%
The needs for training of AM equipment operators	1%
Others	1%

**Table 7:** Distribution of the occupied population in Spain by level of automation. Table prepared using figures from (**) the National Statistics Institute (INE) on population for the first quarter of 2018 [93] and (*) information on the probability of automation and the distribution of the occupied population by level of automation taken from BBVA Research [86]. Own production.

Sector of Activity	Occupied Population According to Industry. Both Genders.		Probability of the Distribution of the Occupied Population per Automation Level (%) (*)		Probability of the Distribution of the Occupied Population Per Automation Level (Absolute Value) (Thousands of Persons)		
Status	Absolute value (**)	Percentage (**)	Low	High	Low	Medium	High
Status	2018T1 (thousands of persons)	2018T1 (%)	<0.3	>0.7	<0.3	>0.3 and <0.7	>0.7
A Agriculture, farming, forestry, and fisheries	833.8	4.4	0.5	10.3	4.2	743.7	85.9
B Extractive industries	34.3	0.2	0.1	0.2	0.0	34.2	0.1
C Manufacturing industry	2420.7	12.8	7.6	18.7	184.0	1784.1	452.7
D Energy supply (electricity, gas, steam, and air conditioning)	73.8	0.4	0.6	0.2	0.4	73.2	0.15
E Water supply and treatment, waste management	147.6	0.8	0.3	0.8	0.4	146.0	1.2
F Construction	1151.9	6.1	4.4	5.3	50.7	1040.2	61.1
G Wholesale and retail trade; motor industry	2934.2	15.5	16.2	21.3	475.3	1833.9	624.9
H Transport and storage	958.5	5.1	1.2	4.2	11.5	906.7	40.3
I Hotel and catering trade	1558.5	8.3	2.6	16.3	40.5	1263.9	254.0
J Information and communications	587.2	3.1	4.9	0.8	28.8	553.7	4.7
K Financial and insurance activities	453.1	2.4	2.1	2.5	9.5	432.3	11.3
L Real estate activities	136.0	0.7	0.3	0.4	0.4	135.0	0.5
M Professional, scientific, and technical activities	974.3	5.2	9.0	2.6	87.7	861.3	25.3
N Clerical activities and ancillary services	916.9	4.9	1.3	3.0	11.9	877.5	27.51
O Public Administration and defense; Social Security	1327.1	7.0	9.8	6.5	130.1	1110.8	86.3
P Education	1323.0	7.0	17.8	1.6	235.5	1066.3	21.2
Q Healthcare activities and social services	1571.3	8.3	16.5	2.6	259.3	1271.2	40.8
R Artistic, leisure, and entertainment activities	373.6	2.0	2.7	1.7	10.1	357.2	6.3
S Other services	459.0	2.4	1.3	1.0	6.0	448.4	4.6
T Domestic service activities in households	637.7	3.4	0.7	0.8	4.5	628.1	5.1
U Activities in organizations	1.8	0.0	-	-	-	-	-
Total	18,874.3	100.0	99.9	100.8	1550.7	15,567.7	1754.0

## Acronyms

AENOR	Asociación Española de Normalización y Certificación; Spanish Association for Standardization and Certification
AI	Artificial Intelligence
ALM	Additive Layer Manufacturing
AM	Additive Manufacturing
ASTM	ASTM International “American Society for Testing and Materials”
DDM	Direct Manufacturing, Direct Digital Manufacture
DMD	Direct Metal Deposition
EIDUNED	Escuela Internacional de Doctorado de la Universidad Nacional de Educación a Distancia; International Doctorate School of the Spanish National Distance-Learning University
FDM	Fused deposition modelling
FECYT	Fundación Española para la Ciencia y Tecnología; Spanish System for Science, Technology and Innovation
FFF	Freeform Fabrication
ICTs	Information and Communication Technologies
INE	Instituto Nacional de Estadística; National Statistics Institute
ISO	International Organization for Standardization
TC 261	Technical Committee 261
LOM	Laminated Object Manufacturing
R&D + I	Research, Development, and Innovation
RM	Rapid Manufacturing
RP	Rapid Prototyping
SFF	Solid Freeform Fabrication
SLA	Stereolithography
SLM	Selective Laser Modelling
SLS	Selective Laser Sintering
STEAM	Science, Technology, Engineering, Arts, and Mathematics
UNED	Universidad Nacional de Educación a Distancia; Distance Learning University
